# Control of Initiation of DNA Replication in *Bacillus subtilis* and *Escherichia coli*

**DOI:** 10.3390/genes8010022

**Published:** 2017-01-10

**Authors:** Katie H. Jameson, Anthony J. Wilkinson

**Affiliations:** 1 Institute of Integrative Biology, University of Liverpool, Crown Street, Liverpool L69 7ZB, UK; katie.jameson@liverpool.ac.uk; 2 Structural Biology Laboratory, Department of Chemistry, University of York, York YO10 5DD, UK

**Keywords:** initiation of DNA replication, DnaA, *oriC*, regulation of DNA replication, *Bacillus subtilis*, sporulation

## Abstract

Initiation of DNA Replication is tightly regulated in all cells since imbalances in chromosomal copy number are deleterious and often lethal. In bacteria such as *Bacillus subtilis* and *Escherichia coli*, at the point of cytokinesis, there must be two complete copies of the chromosome to partition into the daughter cells following division at mid-cell during vegetative growth. Under conditions of rapid growth, when the time taken to replicate the chromosome exceeds the doubling time of the cells, there will be multiple initiations per cell cycle and daughter cells will inherit chromosomes that are already undergoing replication. In contrast, cells entering the sporulation pathway in *B. subtilis* can do so only during a short interval in the cell cycle when there are two, and only two, chromosomes per cell, one destined for the spore and one for the mother cell. Here, we briefly describe the overall process of DNA replication in bacteria before reviewing initiation of DNA replication in detail. The review covers DnaA-directed assembly of the replisome at *oriC* and the multitude of mechanisms of regulation of initiation, with a focus on the similarities and differences between *E. coli* and *B. subtilis*.

## 1. Introduction

The initiation of DNA replication is highly regulated and tightly coupled to the progression of the cell cycle to ensure that the frequency of initiation appropriately matches that of cell division. In this way, cells maintain correct chromosome copy number and ensure success in reproduction [1,2,3]. Under-replication leads to cells likely to be missing essential genetic information, whilst over-replication is highly disruptive of genetic regulatory processes and is frequently associated with disease and cell death.

Regulation of DNA replication is exerted primarily at the initiation step when an initiator protein binds to the origin of replication and promotes the assembly of a nucleoprotein complex from which replication forks diverge [4]. Much of our current understanding of DNA replication and its regulatory control in bacteria is derived from studies of the Gram-negative organism *Escherichia coli*, in which the initiator protein is DnaA and the origin is *oriC*. It is now clear that while the principles underlying the regulation of DNA replication initiation in *E. coli* apply to many other bacteria, the regulatory components are somewhat restricted in their distribution [2,5]. Thus the Gram-positive organism *Bacillus subtilis* has no known DNA replication regulators in common with *E. coli*, moreover, its bipartite origin of replication is strikingly different in arrangement to the continuous origin of *E. coli* [6]. Furthermore, when starved of nutrients, additional layers of DNA replication control are exerted in *B. subtilis* as it enters into the pathway of sporulation which is characterized by asymmetric cell division, and compartment-specific gene expression. 

This review describes our current understanding of DNA replication initiation and its regulation in *B. subtilis*. As bacterial DNA replication is best understood in *E. coli*, we provide an overview of the replication phases of initiation, elongation and termination in this organism before highlighting differences that are known in *Bacillus*. This is followed by an in-depth coverage of initiation of DNA replication including the initiation machinery and the mechanisms of DnaA assembly at the origin, with particular emphasis on the roles of the *Bacillus*-specific components, DnaB and DnaD, in replisome assembly. Next, we discuss the activities of the regulators, YabA and Soj/Spo0J, during growth and Spo0A/Sda and SirA during sporulation. Finally their mechanisms of action are compared with those of the *E. coli* regulatory components. This review is concerned with the regulatory mechanisms of DNA replication initiation in *B. subtilis* and *E. coli*—it is not intended as a comprehensive review of the DNA replication mechanisms of all bacterial species.

## 2. DNA Replication

The process of DNA replication can be separated into three distinct phases: initiation, elongation and termination. During the initiation phase, a nucleoprotein complex assembles at the origin of replication. This induces localized DNA unwinding leading to helicase loading and recruitment of a full complement of replisome machinery. In the elongation phase, this replication machinery carries out template-directed DNA synthesis. This is continuous and processive on the leading strand, but discontinuous on the lagging strand where a more complex cycle of primer synthesis, strand elongation and fragment ligation takes place. Finally, during termination, DNA polymerization is halted at a specific termination site. Regulation of DNA replication occurs principally at the initiation stage, during or prior to the recruitment of the replication machinery. 

### 2.1. Initiation of DNA Replication

**Figure 1:**
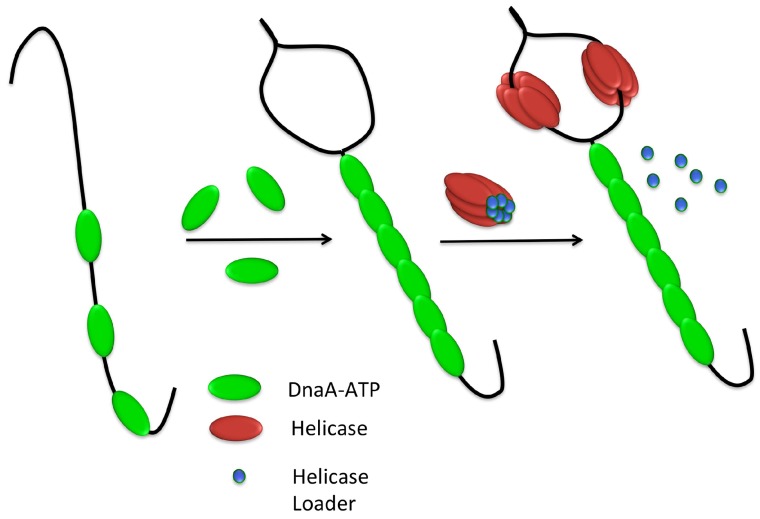
DNA replication initiation at *oriC*: DnaA (**green**) recognizes binding sites on *oriC*, forming a nucleoprotein complex which induces unwinding at the DNA unwinding element (DUE). The helicase loader then facilitates binding of the DNA helicase (**red**) as a prelude to recruitment and assembly of other components of the replication machinery. Figure inspired by [4].

In bacteria, DNA replication is initiated by the binding of a protein initiator, DnaA, to the origin of replication, *oriC* (Figure 1). DnaA is understood to form a right-handed helical oligomer on the DNA [7,8] directed by its binding to a series of recognition sites within the origin termed DnaA-boxes [9]. The formation of this oligomer induces a localized unwinding of the DNA duplex within the origin at an AT-rich region termed the DUE (*D*NA *U*nwinding *E*lement) [10,11]. DnaA then plays a role in recruiting the processive DNA helicase, named DnaB in *E. coli* or DnaC in *B. subtilis* [12], which is loaded onto the unwound single-stranded DNA (ssDNA) by a helicase loader, named DnaC and DnaI respectively [13] (Table 1). 

The helicase subsequently recruits the primase, DnaG, and the polymerase β-clamp, DnaN, which in turn recruits other components of the replication machinery in readiness for de novo DNA strand synthesis [14]. In *B. subtilis*, initiation requires two additional essential proteins, DnaD and DnaB [15], both of which possess DNA remodelling activities [16] and bind to the origin prior to helicase loading [17]. DnaD is thought to play a role in double-stranded DNA (dsDNA) melting, while DnaB appears to have a role in helicase loading. The essential components of the *B. subtilis* DNA replication machinery and their *E. coli* equivalents are listed in Table 1. 

### 2.2. The Elongation Phase

**Figure 2:**
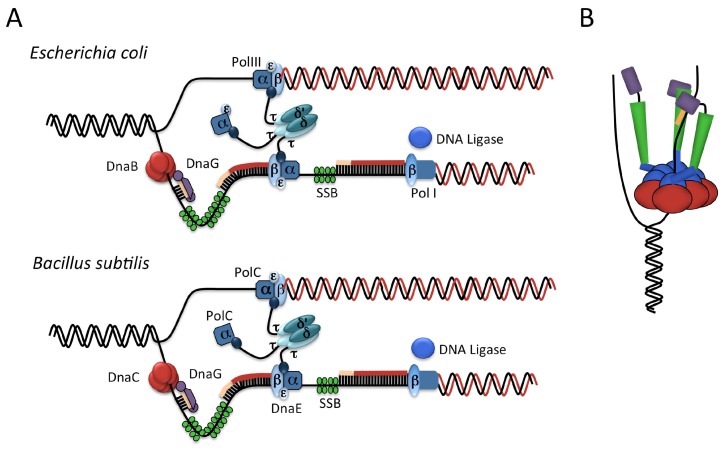
(**A**) Schematic representation of the *E. coli* and *B. subtilis* replisomes showing locations of the helicase, primase, DNA polymerase, the β-clamp and the clamp loader (τ_3_δδ’) at the replication fork. Figure adapted from [20] (**B**) Schematic of primase function. The helicase (**red**) unwinds the parental DNA, positioning a single strand ready for primer formation. The RNA polymerase binding domain (**green**) of one primase molecule forms a complex with the Zn binding domain (**purple**) of another primase molecule and single-stranded DNA (ssDNA) in order to synthesize the primer (**orange**). The C-terminal helicase-binding domain of the primase is shown in blue. Adapted from [24]

During the elongation phase of DNA replication, DNA is synthesized processively by the action of a large multi-subunit complex known as the replisome (Figure 2A). Based on single molecule biophysics studies in *E. coli*, the replisome consists of three DNA polymerase complexes, a hexameric DNA helicase, DNA primase (assumed from structural studies to comprise three subunits [18]), three processivity clamps, DnaN (two of which are associated with the core replisome), and a pentameric clamp loader complex [19]. 

The helicase forms a homohexameric ring that is understood to sit at the head of the replication fork on the lagging strand of the template DNA. The helicase mechanically separates dsDNA by translocating along the lagging template strand in a process driven by ATP-hydrolysis. Separated DNA strands are coated in single-stranded DNA binding protein, SSB, which prevents the strands from re-annealing and offers protection to the ssDNA from nucleases [20,21,22].

The primase, DnaG, contains three functional domains; an N-terminal zinc-binding domain (ZBD), a central RNA polymerase domain (RPD) and a C-terminal helicase binding domain. Three DnaG molecules associate with the N-terminal domains of the helicase, positioned such that the primase captures the ssDNA which has been newly unwound by the helicase, ready for primer synthesis [23,24] (Figure 2B). The primase contains a groove that is thought to interact non-specifically with ssDNA, allowing the primase to track along the ssDNA and orientate it correctly for entry into the active site in the RPD, where primers are synthesized from available ribonucleoside tri-phosphate (rNTPs). The newly synthesized primer is extruded on the outside of the DnaB-DnaG complex, ready for handoff to SSB and DNA polymerase [23]. Whilst the RPD contains the catalytic site for RNA primer synthesis, the ZBD is responsible for modulating the activity of the RPD. Interestingly, the ZBD of DnaG regulates the RPD of an adjacent subunit in *trans* [25]. The RPD and ZBD from separate chains recognize the ssDNA template and initiate primer synthesis at specific trinucleotide recognition sites; with the ZBD increasing the catalytic activity of the *trans* RPD, as well as restricting processivity and primer length [25].

Strand extension in *E. coli* (Figure 2A) is carried out by DNA polymerase III (Pol III), which has an αεθ structure, where α is the catalytic subunit, ε is responsible for proofreading and θ is a non-essential subunit thought to stimulate the activity of ε. Pol III extends the primer with the assistance of the processivity clamp, DnaN (also known as the β-clamp). DnaN sits directly behind Pol III, as a closed ring on the DNA formed from two C-shaped subunits. DnaN binds across, rather than within, the major and minor grooves of duplex DNA, allowing the protein to slide along the DNA. In this way, the β-clamp enables the polymerase to synthesize up to 1000 bases a second [20,21,22]. The synthesis of each lagging strand Okazaki fragment requires the loading of a new β-clamp; thus the clamp loader complex forms part of the replisome machinery. The clamp loader is a pentameric complex with a subunit structure τ_3_δδ’. The τ subunit, the product of the gene *dnaX*, interacts with both the DNA helicase and Pol III—it is thought to play an architectural role at the replisome and couple DNA unwinding and DNA extension [20,21,22].

Elongation in *B. subtilis* occurs by a similar mechanism; however it uses two different, but related, replicative DNA polymerases, PolC and DnaE (Figure 2A). DnaE is more closely related to *E. coli* Pol III than PolC [26]. Both polymerases have been shown to be essential for lagging strand synthesis, whilst PolC is required for leading strand synthesis [27]. Each can extend DNA primers, but DnaE alone is able to extend the RNA primers produced by DNA primase. It is thus thought that DnaE extends the RNA primers with DNA before handing over to PolC for further strand synthesis [27]. This is analogous to systems in eukaryotes where DNA polymerase α extends RNA primers with DNA, before handing over to the lagging strand polymerase δ [20]. 

### 2.3. Termination of DNA Replication

The termination of DNA replication occurs at a termination locus positioned directly opposite *oriC.* In both *B. subtilis* and *E. coli*, replication termination is controlled by a polar mechanism in which the *Ter* site can be approached from a ‘permissive’ or ‘non-permissive’ direction. However, different mechanisms have evolved in each species.

**Figure 3:**
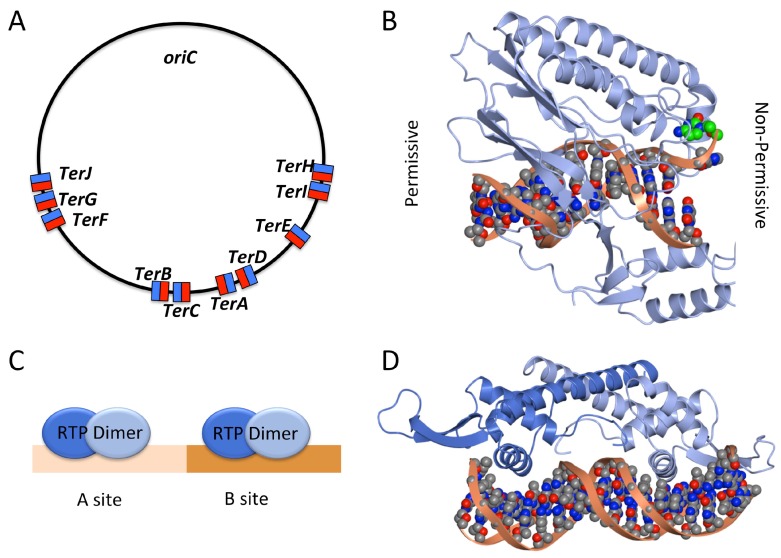
(**A**) Location and orientation of *Ter* sites in *E. coli*: permissive face shown in blue, non-permissive face shown in red; (**B**) Structure of the Tus-*Ter* complex (PDB code: 2EWJ) showing the permissive face (**left**) and the non-permissive face (**right**). On the non-permissive face a specific cytosine base (**green**) flips into Tus when double-stranded DNA (dsDNA) is unwound by the oncoming replication fork, creating a ‘locked’ complex; (**C**) Schematic image of two RTP dimers binding at the A and B sites of the *Bacillus* terminus region; (**D**) Structure of an RTP dimer bound to dsDNA (PDB code: 2EFW) with the sequence of a B-site region; one molecule displays a ‘wing up’ conformation (adjacent to the A site) and the other a ‘wing down’ conformation. (B), (D) and subsequent structural figures were rendered in CCP4MG [34].

In *E. coli*, the locus directly opposite *oriC* is flanked on either side by five non-palindromic 23-bp sites, *Ter*A-J (Figure 3), which bind the monomeric protein Tus (terminator utilisation substance) [28,29,30]. The orientation of these *Ter* sites dictates whether or not a travelling replication fork is able to pass the site or is halted in DNA replication [28,30]. Thus, a replication fork can bypass a *Ter* site unimpeded when travelling in the permissive direction, but is blocked when travelling in the non-permissive direction. For example, in Figure 3A, a replication fork travelling clockwise would bypass *TerH*, *TerI*, *TerE*, *TerD* and *TerA*, but would be halted at *TerC* (or failing that, at *TerB*, *TerF*, *TerG* or *TerJ*). Tus is a 36 kDa protein which specifically binds *Ter* sites in an asymmetric manner [31] (Figure 3B). Collision with the DNA helicase DnaB approaching from the permissive direction, causes Tus to rapidly dissociate. In contrast, when the approach is from the non-permissive direction, Tus-*Ter* forms a roadblock which prevents the translocation of DnaB and the associated replication fork [32]. Tus functions like a ‘molecular mousetrap’ at *Ter*. The trap is set by asymmetric binding of Tus to dsDNA in the non-permissive orientation, such that strand unwinding by the oncoming replication machinery ‘triggers’ the trap causing a specific cytosine base at position 6 of the *Ter* site to flip into a binding site on Tus. This gives rise to a ‘locked’ Tus-*Ter* complex (Figure 3B) which presents a roadblock to the progression of the replication fork [32,33]. 

In *B. subtilis*, the binding of two homodimers of the replication termination protein (RTP) at ‘A’ and ‘B’ sites within the *Ter* region is required to arrest replication (Figure 3C) [35,36]. The approach of the replication machinery from the ‘B’ site results in termination of replication (non-permissive direction) whilst approach from the ‘A’ site allows replication to continue (permissive direction). The crystal structure of a single RTP dimer bound to the native ‘B’ site has been shown to display asymmetry in the ‘wing’ region of the winged-helix domain [37] (Figure 3D). The protomer that lies proximal to the A-site shows a ‘wing-up’ conformation, while the other protomer displays a ‘wing down’ conformation, each making different contacts with the dsDNA. It is possible that this asymmetry gives rise to the ‘permissive’ and ‘non-permissive’ directions. However, A-site binding is also required to block replication fork progression, and A-site binding by RTP is co-operative following B-site binding [38]. The structural consequences of A-site binding are unknown and therefore the molecular basis of RTP action in replication termination remains unknown. Although the details of the *E. coli* and *B. subtilis* replication termination mechanisms vary, they appear to have evolved conceptually similar mechanisms for terminating replication in a direction specific manner. 

## 3. Initiation of DNA Replication

### 3.1. Replication Origins

Replication origins have formed the topic of comprehensive recent reviews [39,40]. Knowledge of replication origins and how they encode DnaA-origin binding is key to the understanding of initiation mechanisms and how they are regulated. All origins harbor sequences that direct the formation of replication complexes, DNA unwinding, and species-specific regulatory activities. Conserved features of all bacterial replication origins include DnaA-box clusters and an AT-rich DUE. However across species, origins vary significantly in organization and length, including the number and spacing of DnaA-boxes and DnaA-box location with respect to the DNA unwinding elements. Of particular relevance to this discussion are two key differences between the origins of *B. subtilis* and *E. coli*: the genomic context of the origin and the number of intergenic regions that constitute *oriC.*

#### 3.1.1. Genetic Context of Replication Origins

The location of the replication origin and its gene context are well conserved across bacterial species, with most flanked by, or containing, the *dnaA* gene [6,41]. The genes surrounding *oriC* and *dnaA* are also well conserved, consisting of the gene cluster *rnpA-rpmH-dnaA-dnaN-recF-gyrB-gyrA* with *oriC* residing in one or two intergenic regions adjacent to *dnaA* [6]. Unusually, the *E. coli* origin has undergone a major rearrangement resulting in a translocation of the origin 44 kb away from the *dnaA* gene and the *rnpA-rpmH-dnaA-dnaN-recF-gyrB-gyrA* cluster [41] so that it is instead flanked by the genes *gidA* and *mioC* [39]. Thus the origin of replication in *B. subtilis* may be more primitive than that of *E. coli*. Moreover, *B. subtilis* may provide a better model for bacterial replication origins in general [6].

#### 3.1.2. Continuous and Bipartite Origins

**Figure 4:**
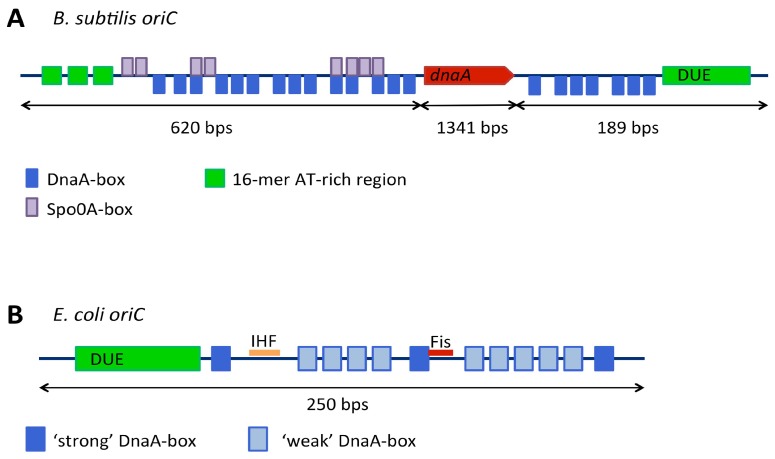
(**A**) *B. subtilis* origin of replication: DnaA-boxes are shown in blue, the *dnaA* gene in red, DNA unwinding element in green and Spo0A-boxes in purple; (**B**) The *E. coli* origin of replication: strong DnaA-boxes are shown in dark blue, weak DnaA-boxes in light blue, the DNA unwinding element in green and binding sites for accessory proteins integration host factor (IHF) and Fis in orange and red, respectively.

Origins are described as either continuous or bipartite according to whether all of the functional elements are contained in one or two intergenic regions respectively. For example, the origin of DNA replication in *B. subtilis* (Figure 4A) is bipartite, containing two DnaA-box clusters, separated by the *dnaA* gene [42,43]. In *E. coli*, the origin of replication is a continuous ≈250 bp element. (Figure 3B). The bipartite origin in *B. subtilis* has been shown to be important for proper replication initiation [36], although it is not clear how this difference in origin structure affects the assembly and architecture of the initiation machinery at the origin. During replication initiation, *B. subtilis oriC* forms looped structures which are thought to be a consequence of the bipartite nature of its origin [44]. These looped structures can also form using *E. coli* DnaA but *E. coli* DnaA is unable to unwind the *B. subtilis* origin. This supports the idea that a mechanism of DnaA binding at the origin leading to DnaA oligomerisation is applicable across bacterial species, as might be expected given the high conservation of DnaA. However, specific assembly and regulation of initiation encoded by each origin is likely to be species-specific.

### 3.2. The DNA Replication Initiator, DnaA

**Figure 5:**
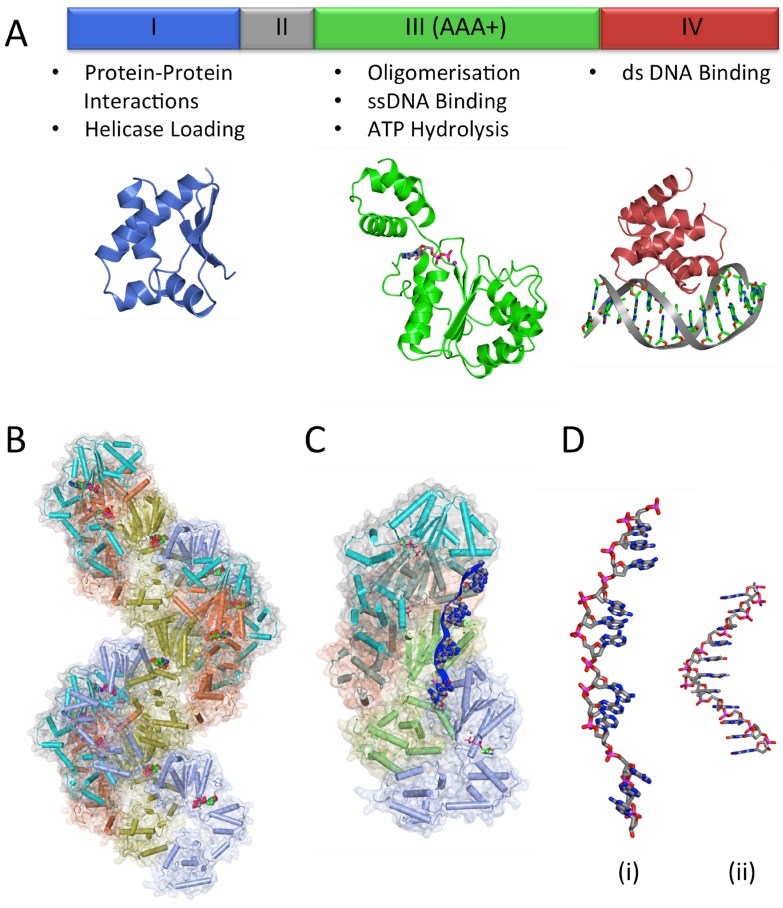
(**A**) Schematic of DnaA showing domain architecture and structures. Domain I (PDB code: 4TPS) is shown in blue, domain II in gray, domain III (PDB code: 1L8Q) in green and domain IV (PDB code: 1JLV) in red. Figure adapted from [2]. (**B**) DnaA Domains III–IV bound to a non-hydrolysable ATP analogue (PDB code: 2HCB) form a spiral structure that is thought to mimic DnaA oligomerisation at the origin. A repeating pattern of DnaA protomers is shown in light blue, gold, coral and cyan; (**C**) ssDNA-binding mode of DnaA domains III–IV (PDB code: 3R8F). Separate DnaA protomers are shown in light blue, green, gray and cyan; (**D**) ssDNA binding by DnaA stretches the strand into an extended form (i) compared to B-form DNA (ii).

The initator DnaA is a member of the AAA+ ATPase family (ATPases associated with diverse cellular activities) and contains four distinct domains [45,46] (Figure 5A). In the cell, DnaA exists in both ATP- and ADP-bound forms [47]. DnaA–ATP is considered to be the ‘active’ form of the protein as this is required for oligomerisation at the origin [48,49], an event which triggers DNA unwinding and ultimately, assembly of the replisome. The C-terminal domain IV of DnaA is a dsDNA binding domain which is responsible for DnaA-box recognition [50,51]. The adjacent domain III contains Walker A and B motifs that are involved in ATP binding and hydrolysis. This domain plays a role in self-interaction/oligomer formation and in ssDNA binding [52]. Domain II of DnaA is poorly conserved and of variable length and considered to form a flexible linker which may play a role in controlling replication efficiency [53]. Finally, the N-terminal domain I is an ‘interaction domain’ which has been shown to interact with various protein regulators of DnaA across different organisms [54,55,56]. In *E. coli*, it also interacts with the helicase, DnaB [57], and has been suggested to play a role in the self-assembly of DnaA at the origin.

#### 3.2.1. DnaA-Box Recognition by DnaA

The DnaA-boxes within the origin of replication vary in their affinity for DnaA, according to their similarity to a consensus binding sequence, and on the adenosine nucleotide bound state of DnaA [58,59]. In *E. coli* and *B. subtilis*, the consensus DNA-box is the nine-base-pair sequence, 5′-TTATNCACA-3′ [60].

An X-ray structure of DnaA domain IV bound to a consensus DnaA-box sequence revealed that DNA binding is mediated by a helix-turn-helix which interacts primarily with the major groove of the dsDNA, with additional contacts made in the adjacent minor groove [51] (Figure 5A). Base-specific interactions were observed at 8 of the 9 base pairs in the DnaA-box; the exception being the base pair at position 5, where there is no sequence preference [51]. Mutations at residues involved in base-specific interactions result in loss of DnaA-box binding specificity, or loss of DNA-binding altogether [61].

#### 3.2.2. Variable Affinity of DnaA-Boxes

The DnaA-boxes at the origin can be either ‘strong’ or ‘weak’; where strong boxes bind both DnaA–ATP and DnaA–ADP with equal affinity and ‘weak’ boxes have a much greater relative affinity for DnaA–ATP [62]. In order for the helical DnaA oligomer to form at the origin and induce DNA unwinding, both strong and weak DnaA-boxes need to bind DnaA [63,64]. In the *E. coli* origin (Figure 4B), DnaA-boxes are distributed such that three strong boxes lie at either end of the origin and at its centre. As DnaA–ATP recruitment to the origin has been shown to be co-operative, these strong boxes are thought to form anchoring points from which the DnaA oligomer can grow [63,65]. In this model, DnaA–ATP is recruited to weak binding sites via co-operative interactions with DnaA–ATP molecules already bound to neighbouring sites [65].

#### 3.2.3. DnaA Oligomerisation

Domains III–IV of *Aquifex aeolicus* DnaA have been shown to adopt an open spiral conformation [8] which likely mimics the right-handed helical oligomers ATP-bound DnaA forms at *oriC* [7] (Figure 5B). Adjacent protomers interact with one another via two clusters of conserved residues located on either side of the nucleotide binding pocket [8]. Significantly, DnaA–ADP cannot form this right-handed oligomer [7]; instead it appears to be monomeric [66]. The binding of ATP induces a small conformational change in the ATPase domain which allows an adjacent DnaA protomer to interact with the ATP via a conserved arginine residue known as an ‘arginine finger’. This interaction is significant in stabilising the DnaA helical filament [8] and similar ‘arginine finger’ interactions are frequently observed in other AAA+ ATPases [67]. Significantly, these observations provide a molecular explanation for why DnaA–ATP is the ‘active’ form of the initiatior.

In order to reconcile the Domain IV-DnaA-box binding mode with the DnaA helical oligomer formed by DnaA domains III–IV on dsDNA, a conformational change in the linker helix between domains III and IV has been invoked [51,68]. A significant kink in the linker helix is observed in the ATP-bound structure compared to the ADP-bound form (where the helix is straight) suggesting that the two domains are conformationally uncoupled and would be able to rotate with respect to one another to allow filament formation at the origin [8].

#### 3.2.4. DNA Unwinding and ssDNA Binding

After the DnaA oligomer has formed at the origin, localized strand unwinding occurs at the DUE [69] (Figure 1). Based on structural work carried out with *Aquifex aeolicus* DnaA, unwinding is mediated by the DnaA-oligomer, which introduces positive writhe in the bound DNA [7]. Compensatory negative writhe at the DUE would facilitate DNA unwinding [4,8]. This unwound DNA is then stabilized by binding to the ssDNA binding site of DnaA located in the ATPase domain [69,70]. ATP-bound DnaA binds ssDNA in the same open spiral conformation displayed by DnaA domains III-IV [69]. In complexes of DnaA with single-stranded poly-(dA) DNA, each DnaA protomer binds three nucleotides, making multiple interactions with the DNA phosphodiester backbone. Each nucleotide triplet displays a normal B-form DNA conformation, but the triplets are separated by gaps of approximately 10 Å creating an overall extended form of DNA [69] (Figure 5D). This strand extension has been shown to be ATP-dependent in solution and is highly reminiscent of ssDNA binding displayed by the homologous recombination protein, RecA. The third base of each triplet is rotated however, making bases in the DnaA-bound strand discontiguous; this presumably prevents re-annealing of the strand at the origin [69] (Figure 5D).

Recently identified trinucleotide sequences within bacterial origins termed ‘DnaA-trios’ appear to be responsible for providing specificity of binding of DnaA to ssDNA, facilitating DNA-unwinding at the origin [71]. The trinucleotide motifs have the consensus sequence 3′-G/AAT-5′ and are separated from a proximal DnaA-box, or pair of boxes, by a GC-rich region. A DnaA molecule bound to the proximal DnaA-box via domain IV appears to be able to bind to the first of these DnaA-trio motifs via its AAA+ motif in domain III. Additional DnaA molecules interact with further DnaA-trio motifs, forming an oligomer on the ssDNA and facilitating DNA-unwinding [71].

#### 3.2.5. *Bacillus* DnaA

*B. subtilis* DnaA has also been shown to form helical oligomers on both double and single stranded DNA [72], moreover, the DnaA–ATP form is required for co-operative binding to the origin [73]. *Bacillus anthracis* DnaA displays an ATP-dependent variable affinity for DnaA-box sequences [74]. Together these findings imply *Bacillus* DnaA functions at *oriC* in a similar manner to *E. coli* DnaA.3.2.6. The Role of DnaA Domains I–II in Initiation

DnaA domains I–II are not necessary for DnaA oligomerisation, or DnaA loading onto ssDNA [71]. Nevertheless DnaA domains I–II are required for initiation of replication [75]. DnaA domain I is known to interact with several regulators of DNA replication initiation; these include *E. coli* DiaA [76] and *H. pylori* HobA [55]—structural homologues and promoters of initiation in their respective organisms— and *E. coli* Hda [77] and *B. subtilis* SirA—two negative regulators of initiation [54]. In *E. coli*, domain I also interacts with the helicase DnaB [57,78,79] where it is thought to help correctly orientate the loading of DnaB at the origin. Domain I of DnaA has also been suggested to play a role in the self-assembly of DnaA at the origin [80,81]. It has a K homology (KH)-domain fold typically found in ssDNA binding proteins [82,83,84] (Figure 5A). In vitro DnaA domain I binds to single-stranded *oriC* DNA, albeit weakly, suggesting a potential role in binding ssDNA at the origin [84]. However, no ssDNA binding role has yet been demonstrated for DnaA domain I in vivo.

Domain II has been shown to be unstructured, consistent with a role as a flexible tether between domains I and III. It is not completely dispensable for DnaA function, but it is poorly conserved and varies significantly in length between organisms [46]. Two studies in *E. coli* have indicated that domain II contributes to the efficiency of initiation of replication. In one study, a spontaneous deletion in domain II allowed suppression of an over-initiation phenotype, suggesting that the deletion had reduced the efficiency of DNA replication initiation [85]. In another study, when deletions longer than 17–19 residues were made from domain II, the doubling time of cells harbouring this mutation was increased compared to wild type cells, suggesting the length of domain II contributed to the efficiency of DNA replication [53]. The same study defined the minimum length of domain II in *E. coli* to be 21–27 residues [53].

### 3.3. Helicase Loading

**Figure 6:**
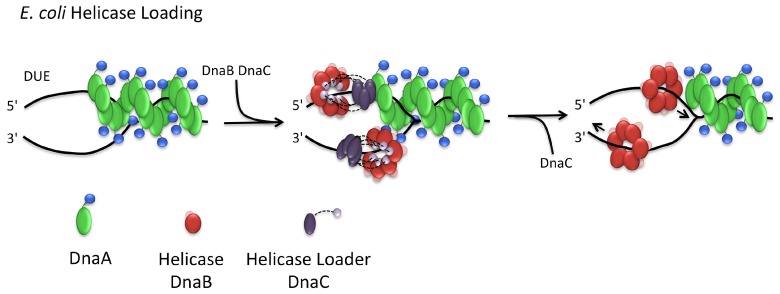
In *E. coli*, the initiator DnaA forms a helical oligomer during initiation which associates with the upper strand of the ssDNA. Following unwinding of the DUE, interactions between DnaA and DnaB or DnaC in the DnaC–DnaB complex are thought to correctly orientate DnaB for loading onto the bottom and top strands of DNA, respectively. Figure adapted from [88].

Following the unwinding of the DUE, a homohexameric DNA helicase is loaded onto single stranded DNA at the replication origin by the action of a helicase loader protein. In *E. coli*, the helicase, DnaB, is loaded onto the ssDNA by the helicase loader DnaC. This occurs via a ‘ring-breaking’ mechanism whereby DnaC forms a spiral oligomer which remodels the hexameric DnaB ring, producing a break in the ring large enough to allow loading onto ssDNA [86]. The recruitment of the DnaB–DnaC complex to the origin occurs by an interaction between the N-terminal domain of DnaA and the helicase, DnaB [12,84,87]. This interaction is thought to orient DnaB for loading onto the bottom strand of the DNA, while an interaction between the AAA+ domains of DnaA and DnaC is thought to recruit the complex in the right orientation for DnaB loading on the upper strand (Figure 6) [86,88].

The primase, DnaG, is next recruited via an interaction with the N-terminal domain of DnaB. Subsequently, active primer formation appears to induce the dissociation of DnaC, in a step which is necessary for DnaB to begin to function as an active helicase. Release of DnaC appears to be dependent on the ATPase activity of DnaC which is thought to be induced by a conformational change in DnaB during primer formation [89]. DnaG interacts with the N-terminal domain of DnaB, while DnaC interacts with its C-terminal domain [90]. The loading of the helicase is important for the recruitment of the DNA polymerase clamp, DnaN. The clamp, in turn, recruits the DNA polymerase, in readiness for primer elongation [90,91].

Helicase loading in *B. subtilis* is thought to occur via a different mechanism known as ‘ring assembly’ [91]. In this model, the helicase loader, DnaI, facilitates the assembly of the helicase DnaC onto ssDNA [92,93]. In the presence of DnaI, pre-formed DnaC hexamers exhibit no helicase or translocase activity in contrast to monomeric DnaC which displays both helicase and translocase activities [92]. The helicase loader DnaI, like *E. coli*’s loader protein, contains an N-terminal helicase interaction domain and a C-terminal AAA+ domain [94]. The ATPase activity of the C-terminal domain of DnaI is stimulated in the presence of ssDNA, but only once inhibition by the N-terminal domain is overcome; binding of the N-terminal domain of DnaI to the helicase DnaC reveals a cryptic ssDNA binding site on the C-terminal domain [93]. It is thought that this then facilitates helicase loading onto ssDNA. Finally, the ATPase activity of the C-terminal domain may stimulate the release of DnaI once loading has occurred [93].

### 3.4. Bacillus Initiation Proteins DnaD and DnaB

Besides DnaA, DnaC (equivalent to *E. coli* DnaB), DnaI and DnaG, DNA replication initiation in *B. subtilis* requires the presence of two additional essential proteins, DnaD and DnaB [95,96]. A summary of their structure and function forms part of the discussion in an excellent recent review [6]. Both DnaD and DnaB are components of the replication initiation machinery at *oriC* [15] as well as components of the replication restart machinery which is DnaA-independent [97]. Both proteins exhibit DNA remodelling activities [16] and share structural similarity [96]. The *B. subtilis* initiation machinery assembles in a hierarchical manner, and DnaD and DnaB recruitment occurs between DnaA binding at *oriC* and the loading of the helicase, DnaC [17]. On binding to *oriC*, DnaD forms direct interactions with DnaA [98]. DnaD is required for the recruitment of DnaB and this, in turn, is then required for recruitment of DnaC-DnaI [17]. Together DnaB and DnaI are thought to function as a helicase loader [92]. 

The exact roles of DnaD and DnaB in replication initiation remain unclear. DnaD is able to untwist supercoiled DNA into an open looped form [99]. It forms tetramers which can assemble into large protein scaffolds that appear to mediate DNA loop formation and enhance melting of dsDNA [100]. The N-terminal domain of DnaD (DDBH1) is implicated in tetramer formation [101,102] with the C-terminal domain (DDBH2) involved in both double- and single-stranded DNA binding [100,102]. The full-length protein is required for DnaD to exhibit DNA looping and melting activities [100,102]. It is estimated that there are 3000–5000 DnaD molecules [103] in the cell and this relative abundance has led to the suggestion that DnaD plays a global role in DNA remodeling, beyond that required for DNA replication initiation [16]. In support of this idea, a study has shown that DNA remodeling by DnaD stimulates DNA repair by Nth endonucleases in response to DNA damage following treatment with H_2_O_2_ [104].

It is generally thought that DnaB acts together with DnaI to enable the loading of DnaC onto forked DNA [92]. However, studies [93,105] suggest that DnaI alone is sufficient to load the helicase onto DNA and that DnaB is required to recruit DnaC–DnaI to the origin [17] and that it acts to stimulate the helicase and translocase activities of DnaC in the presence of DnaI [92]. DnaB has also been implicated in the association of the DNA replication machinery with the cell membrane [95,106]. It has also been shown to laterally compact DNA—although it is not known how this contributes to its function [16].

**Figure 7:**
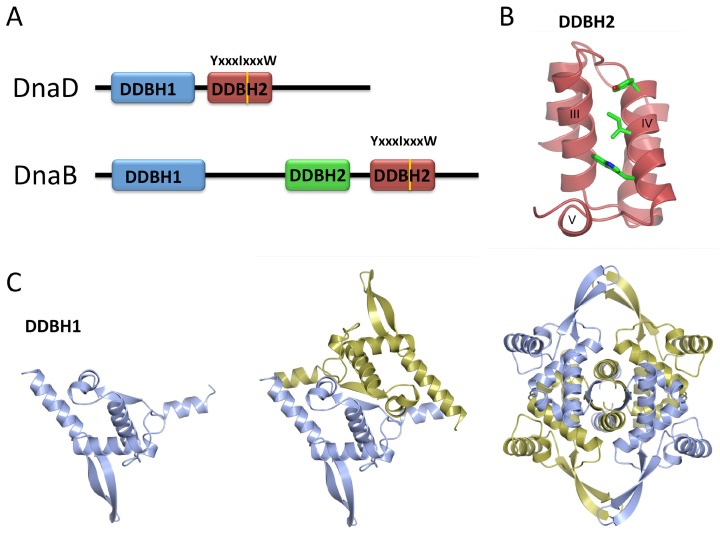
(**A**) Diagram showing the architecture of DnaD and DnaB. Conserved DNA binding motif YxxxIxxxW is marked on the relevant DDBH2 domain; (**B**) Ribbon diagram of the DnaD DDBH2 domain from *Streptococcus mutans* (PDB code: 2ZC2). Tyrosine, Isoleucine and Tryptophan residues of the YxxxIxxxW motif are coloured by atom (carbon in green, nitrogen in blue and oxygen in red); (**C**) Ribbon diagram of DnaD DDBH1 domain from *Bacillus subtilis* (PDB code: 2V79) showing a winged helix with additional structural elements. Monomer, dimer and tetramer architectures are shown. Dimer and tetramer interactions are mediated by the β-strand of the additional helix–strand–helix. Figure inspired by [6].

Although DnaD and DnaB show little sequence similarity, a Hidden Markov Model analysis identified two shared domains known as DDBH1 and DDBH2 (DDBH2 belongs to the PFAM domain: *DnaB*_2). DnaD has a DDBH1–DDBH2 architecture, whilst DnaB has a DDBH1–DDBH2–DDBH2 organization [96] (Figure 7A). The structure of the DDBH1 domain of DnaD revealed a winged helix domain with two additional structural elements: an N-terminal helix–strand–helix and a C-terminal helix [101] (Figure 7C). The β-strand of the helix–strand–helix was found to mediate interactions between DnaD molecules in both dimer and tetramer formation (Figure 7C). The C-terminal helix has been shown to be important in higher-order oligomerisation of these tetramers [101]. These structural elements appear to be present in DnaB DDBH1 [96] which has also been shown to form tetramers mediated by its N-terminus [16], suggesting that DnaB and DnaD share similar oligomerisation properties.

DnaD’s DDBH2 domain has been shown to be involved in DNA-binding and in DNA-dependent higher-order oligomerization [102]. Two structures of the DDBH2 domain of DnaD homologues from *Streptococcus mutans* (PDB code: 2ZC2) and *Enterococcus faecalis* (PDB code: 2I5U) show a compact helical structure with four longer helices I–IV and a shorter fifth helix (V) of only 4 residues (Figure 7B). Although residues following helix V are poorly conserved across DnaD homologs, secondary structure prediction and analysis of the *B. subtilis* DnaD DDBH2 domain by NMR suggests that helix V is extended by a further seven residues [96]. Helix V is followed by a region at the C-terminus that is predicted to be disordered. A YxxxIxxxW motif residing in helix IV, the poorly conserved helix V and the C-terminal unstructured region [96] have been shown to be important for ssDNA binding. These structural elements appear to be conserved in the second of the DDBH2 domains of DnaB [96]. This domain has been implicated in dsDNA and ssDNA binding as well as in higher-order oligomerization [107]. Again, this suggests that the domains play similar roles in the respective proteins.

A DnaB (1–300) fragment encompassing DDBH1–DDBH2 (missing the C-terminal DDBH2 domain) forms tetramers and binds ssDNA [96,107]. Interestingly, C-terminally truncated cytosolic forms of DnaB have been observed during the mid–late growth phase. Full length DnaB alone is observed at *oriC*, thus proteolysis may be regulating DnaB function [107]. It is unclear whether the truncated version of the protein has a discrete function [107], however the different DNA binding capabilities of the DDBH2 domains of DnaB may be important in differentiating the functions of the full-length and truncated versions of DnaB.

### 3.5. Regulation of DNA Replication

#### 3.5.1. During Vegetative Growth in *B. subtilis*

##### YabA

YabA is a negative regulator of DNA replication in *Bacillus subtilis*, affecting both the timing and synchrony of DNA replication in vegetatively growing cells [108]. Deletion of *yabA* causes an increased frequency of initiation events and asynchronous DNA replication [108] as well as a growth phenotype associated with increased initiation events [109,110]. YabA interacts with both the replication initiator, DnaA, and the DNA polymerase clamp, DnaN [109,110]. Mutations of YabA affecting the interaction with either DnaA or DnaN have been shown to exhibit an over-initiation phenotype similar to that in Δ*yabA* cells. This suggests that both interactions are important for replication regulation [110].

Expression of *yabA* genes encoding DnaA-loss-of-interaction or DnaN-loss-of-interaction mutations disrupts the formation of YabA foci at mid-cell, where it is assumed that YabA is co-localized with the replisome. Significantly, however, co-expression of DnaA-loss-of-interaction and DnaN-loss-of-interaction YabA-mutants restores YabA foci, presumably through a hetero-oligomer produced by the two mutants. This implies that both interactions are simultaneously required for YabA localization at the replisome [110,111].

**Figure 8:**
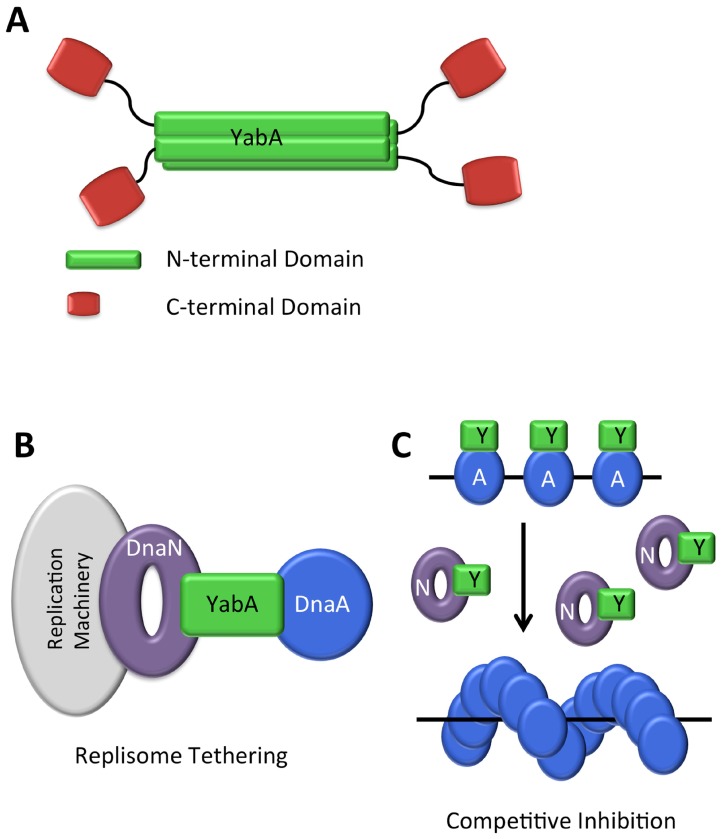
(**A**) Schematic of YabA tetramer structure: YabA N-terminal domains (**green**) form a 4-stranded coiled coil structure. Pseudo-monomeric C-terminal Zn-binding domains (**red**) are attached by flexible linkers; (**B**) Replisome tethering model. YabA tethers DnaA to the replisome via an interaction with both DnaA and DnaN, titrating DnaA away from the replication origin; (**C**) Co-operative inhibition model. YabA (Y) inhibits the cooperative binding of DnaA (A) at the origin during replication. When DnaN (N) is released after replication, YabA binds DnaN, releasing DnaA.

YabA forms tetramers through interactions of N-terminal coiled-coil domains to form an intermolecular 4-helix bundle. This provides a structural scaffold from which four C-terminal Zn-binding domains project. These are connected to the N-terminal domain by a flexible linker and they appear to be independent domains [111] (Figure 8A). The determinants on YabA for DnaA and DnaN interactions lie within these C-terminal domains. Significantly, yeast three-hybrid experiments show that full-length YabA is able to interact simultaneously with DnaA and DnaN [110], whereas the C-terminal domain alone cannot [111]. Thus, the YabA tetramer organization facilitates simultaneous interactions with DnaA and DnaN.

Despite much study, the mechanism of YabA action remains elusive. YabA is not able to promote DnaA–ATP hydrolysis in vitro [112], however, it has been shown to affect the co-operative binding of DnaA to *oriC* [73], and it is capable of disrupting DnaA oligomerisation in vitro [112]. It is not clear, however, if this is its main mode of action in vivo. Two alternative models have been proposed. In the first, YabA tethers DnaA to DnaN at the replisome for most of the cell cycle (Figure 8B) [113], sequestering DnaA from the origin during ongoing rounds of replication. This model is consistent with the alternate localisations of DnaA in wild type and Δ*yabA* cells. In wild type cells, DnaA localizes at the origin in small cells (which are at early points in their cell cycle) and at mid-cell, co-incident with DnaX and therefore the replisome, in larger cells (in later stages of the cell cycle) [113]. In Δ*yabA* cells, by contrast, DnaA is localized with the origin throughout the cell cycle [113].

The alternative model proposes that YabA binds to DnaA at *oriC* so as to inhibit its cooperative binding to further DnaA molecules throughout the cell cycle up to the point where DNA replication is completed and the replisome disassembles. At this point free DnaN competitively titrates YabA away from its complex with DnaA, allowing the latter to bind cooperatively at the origin (Figure 8C) [73]. This model is consistent with evidence that the cellular level of DnaN correlates with the frequency of replication initiation, with increased DnaN levels increasing replication initiation frequency, and decreased levels, decreasing initiation frequency [73]. Additionally, in a strain replicating from a DnaA-independent origin, *oriN*, YabA was shown to affect the cooperativity of DnaA binding at *oriC*, and increased levels of DnaN removed YabA from *oriC*, suggesting that DnaN could be controlling the binding of YabA at the origin.

Further studies to establish the dynamics and stoichiometry of the interactions between YabA, DnaA and DnaN are required to further refine and reconcile these models: bearing in mind that they are unlikely to be mutually exclusive.

##### Soj/Spo0J

Soj is an ATPase which negatively and positively regulates DNA replication in *B. subtilis* [114], according to its oligomeric state [115], which is controlled by nucleotide binding. ATP-bound Soj forms dimers which co-operatively interact with DNA in a sequence unspecific manner, whilst ADP-bound Soj is monomeric [116]. Dimeric ATP-bound Soj appears to stimulate initiation of replication, whilst monomeric Soj inhibits replication [72,115]. Spo0J regulates Soj activity by stimulating its ATPase activity, thus converting the dimer back to the monomeric form [115].

Soj appears to interact with the ATPase domain (III) of DnaA, although it does not affect ATP binding or hydrolysis by DnaA [72]. Instead, it acts by inhibiting DnaA oligomerisation at *oriC*. A Soj mutant trapped in the monomeric state has been shown to inhibit DnaA oligomer formation both in vitro and in vivo [72]. Curiously, Soj trapped in the dimeric state is also able to interact with DnaA on a similar surface, without inhibiting DnaA oligomerization. Thus, it has been suggested that monomeric Soj inhibits conformational changes in DnaA that are needed to form an active initiation complex [72], whilst dimeric Soj may stabilize DnaA in this oligomerization-competent conformation [72].

Soj and Spo0J are orthologues of ParA and ParB, respectively. ParA, and ParB, along with a *cis*-acting DNA sequence parS, are components of a plasmid partitioning system found in many prokaryotic species. These systems ensure partitioning of low copy number plasmids into daughter cells. ParB binds to parS sequences on the plasmid, while ParA forms filaments on chromosomal DNA. An interaction between ParA and ParB simulates the ATPase activity of the former, which is thought to cause dissociation of the terminal ParA molecule from the filament; the plasmid can then either dissociate or translocate along the chromosomal DNA by binding to the next ParA molecule. Continuous cycles of ParA assembly and disassembly lead to equidistribution of the plasmids within the cell [117], ensuring partitioning on either side of the division plane [118].

Chromosomal orthologues of ParA and ParB and parS sites are found in some bacterial species and it is attractive to assume that they perform a role in chromosomal segregation similar to that of the plasmid partitioning proteins. In *B. subtilis*, although Spo0J-parS contributes to accurate chromosome segregation, it is not essential for this function [119]. Instead it plays a role in the recruitment of the SMC complex to the origin, and it is the SMC proteins that are responsible for proper segregation and condensation of the chromosome [120,121]. Regardless, Spo0J provides a mechanism through which *B. subtilis* may be able to co-ordinate DNA replication and chromosome segregation [121].

##### DnaD

DnaD has also been reported to play a role in the regulation of DNA replication initiation in *B. subtilis*. Like YabA, DnaD has been shown to inhibit the ATP-dependent cooperative binding of DnaA to *oriC* DNA [122] and to affect the formation of helical DnaA filaments in vitro [112]. It remains unclear however, how these activities can be reconciled with the role of DnaD in vivo, where it is essential for DNA replication initiation.

##### DnaA-Box Clusters

A *B. subtilis* deletion strain, in which six DnaA-box clusters (DBCs) found outside of the replication origin were removed, displayed an early initiation of DNA replication phenotype. This phenotype was strong only when all six clusters were removed and could be partially relieved by the re-introduction of a single DBC at various locations [123]. Nevertheless, these data suggest that *B. subtilis* DNA replication is sensitive to the amount of free DnaA in the cell, which might otherwise be bound at these sites.

#### 3.5.2. During Sporulation in *Bacillus subtilis*

**Figure 9:**
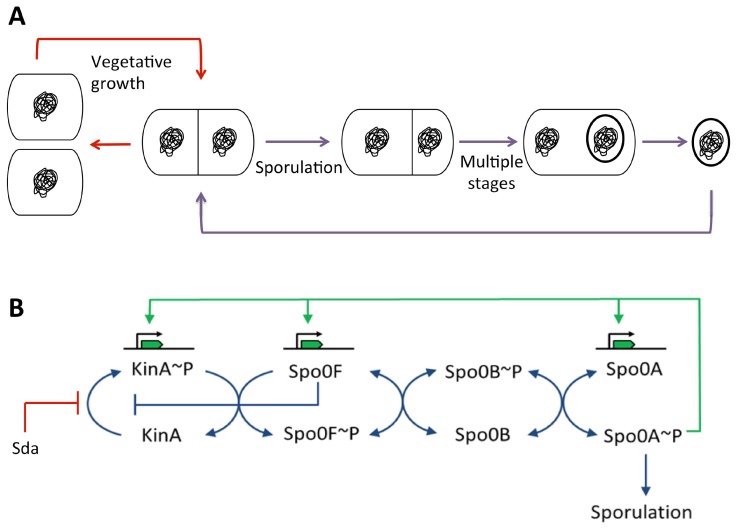
(**A**) Vegetative growth and sporulation in *B. subtilis.* In normal vegetative growth (**red** arrows) cells divide symmetrically, producing identical daughter cells. During sporulation (**purple** arrows) cells divide asymmetrically forming a mother cell and forespore; each receives an identical copy of the genome, and through differential gene regulation they experience different fates. The mother cell engulfs the forespore, nurturing it as it matures. In the final stages, the mother cell lyses releasing the dormant spore; (**B**) Phosphorelay leading to the induction of sporulation. A series of phosphoryl transfer reactions lead to the accumulation of threshold levels of Spo0A~P needed for entry into the sporulation pathway.

A characteristic of *B. subtilis* is its ability to differentiate under nutrient limiting conditions to form a dormant endospore. The spore is metabolically inactive and resistant to harsh conditions such as high temperatures, desiccation and ionizing radiation. When nutrients become available again, the spore can germinate, returning the cell to vegetative growth, even after thousands of years [124,125]. Unlike vegetative growth which is characterized by division at mid-cell, during sporulation the cell divides asymmetrically forming a larger mother cell compartment and smaller forespore compartment (Figure 9A). These two daughter cells each contain an identical copy of the genome, however, differential pathways of gene expression lead to dramatically different cell fates. The forespore is engulfed by the mother cell, and in the cytoplasm of the latter it matures into a resistant spore. In the final stages, the mother cell lyses to release the fully formed spore [126] (Figure 9A). Entry into the sporulation pathway is under the control of a complex signaling pathway, at the heart of which is an expanded two-component system termed the phosphorelay, which culminates in the phosphorylation of the response regulator Spo0A, the master control element of sporulation [127] (Figure 9B). Spo0A~P acts as a transcriptional regulator, controlling directly or indirectly the expression of over 500 genes [128].

At the point of entry into sporulation, DNA replication and asymmetric cell division must be coordinated to ensure that the cell contains two, and only two, copies of the chromosome—one destined for the mother cell and the other for the forespore. Trapping of more than a single chromosome in the forespore compartment can reduce the viability of the spore and its capacity to germinate [129].

##### Spo0A~P Pulsing

It has long been recognized that there is a ‘sensitive period’ in the cell cycle when the cell can enter into the sporulation pathway. If the cell progresses beyond this point, it is committed to a new round of vegetative division [130,131]. The critical determinant is the concentration of Spo0A~P which fluctuates over the course of the cell cycle and is at its highest immediately after DNA replication is completed [129,132]. A threshold level of Spo0A~P must be reached for sporulation to be triggered. As Spo0A~P levels increase, low-threshold target genes are turned on, however, a higher threshold Spo0A~P concentration must be achieved in order to trigger the sporulation process [133,134].

Spo0A~P pulsing is linked to DNA replication, but until recently it was not known how. The cellular Spo0A~P concentration is controlled by the sporulation inhibitor protein, Sda [129]. Sda inhibits the sporulation sensor kinases KinA and KinB, which feed phosphate into the phosphorelay leading ultimately to the phosphorylation of Spo0A [135,136,137] (Figure 9B). Sda production is controlled at the transcriptional level by DnaA [129,135] such that *sda* expression requires the presence of replication active DnaA. Thus, Sda levels spike at the same time as, or just after, the replisome forms [129]. Sda is subsequently rapidly proteolysed [138]. This provides a feedback mechanism whereby Sda blocks phosphorylation of Spo0A and entry into sporulation, during ongoing rounds of DNA replication [129]. However, factors other than Sda influence Spo0A~P pulsing, as deletion of Sda does not prevent the pulsing of Spo0F levels (*spo0F* is a ‘low-threshold’ gene under the control of Spo0A~P) [139] suggesting that Spo0A~P pulsing still occurs.

The chromosomal arrangement of the phosphorelay genes s*po0F* and *kinA* is important for Spo0A~P pulsing [129]. *spo0F* is located close to the replication origin, in contrast to *kinA* which is located near the replication terminus. As a result, two copies of *spo0F* will be present in the cell during most of the period of DNA replication, alongside a single copy of *kinA* [132]*.* Alterations in the chromosomal positioning of *spo0F*, or induction of Spo0F from an inducible promoter, have been shown to affect Spo0A~P pulsing [132], with high Spo0F:KinA ratios inhibiting KinA phosphorylation and preventing sporulation [140,141]. As rapidly growing cells undertake multiple rounds of DNA replication simultaneously, the Spo0F:KinA ratio also provides a mechanism for inhibiting sporulation under nutrient-rich conditions. Collectively, the chromosomal arrangement of the phosphorelay genes in *B. subtilis*, together with direct inhibition of KinA activity by Sda, serve to coordinate the entry into sporulation with DNA replication.

##### SirA

Spo0A~P pulsing provides a mechanism for preventing replicating cells from entering into sporulation. Interestingly, cells which are artificially induced to sporulate under conditions of rapid growth are able to maintain correct chromosome copy number [142]. This is attributable to the activity of SirA, an inhibitor of DNA replication, produced under Spo0A~P control. Deletion of *sirA* results in loss of chromosome number control upon induction of sporulation during rapid growth [142]. Meanwhile, cells overproducing SirA do not form colonies on plates and in liquid culture many of these cells are elongated and anucleate, with some containing nucleoids which have been severed by division septa—a phenotype reminiscent of DnaA depletion [142,143]. SirA inhibits DNA replication through a direct interaction with DnaA [143]. A genetic screen indicated that the determinants of SirA binding reside in domain I of DnaA [54] and a later structure of a complex of SirA with DnaA domain I fully delineated this binding surface [144]. Cells harbouring alleles with *sirA* point mutations mapping to the DnaA domain I binding surface of SirA, exhibit a similar phenotype to Δ*sirA* cells. Moreover, these mutations disrupted SirA foci normally observed in sporulating cells [144]. This suggests that SirA localizes to the replisome via an interaction with DnaA during sporulation.

**Figure 10:**
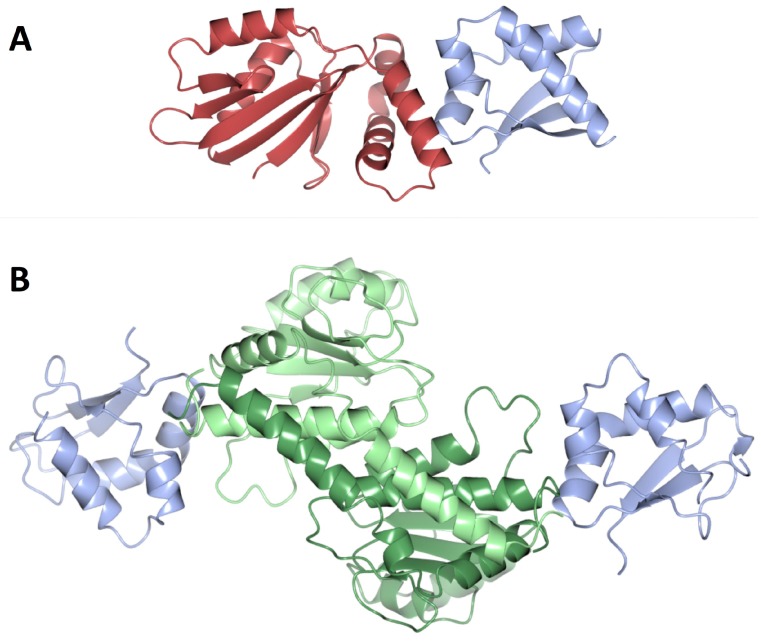
(**A**) SirA–DnaA-DomainI structure (PDB code: 4TPS). SirA is shown in crimson, domain I of DnaA in blue; (**B**) HobA–DnaA-DomainI structure (PDB code: 2WP0). HobA is shown in green, domain I of DnaA in blue. HobA and SirA interact on equivalent surfaces of DnaA, despite exerting different regulatory effects.

Intriguingly, SirA binds to a surface on DnaA domain I structurally equivalent to that used by the positive regulators of DNA replication initiation, DiaA and HobA, structural homologues found in *E. coli* and *Helicobacter pylori*, respectively (Figure 10). This raises an intriguing question about the role of DnaA domain I in replication initiation in the respective organisms. How is the same topological site used to positively and negatively regulate replication initiation?

Recently, SirA was shown to facilitate chromosome segregation during sporulation, independent of its role in DNA replication regulation [145]. Newly synthesized bacterial origins are localized to a cell pole (or future pole) with high fidelity. In sporulating cells, *oriC* must be segregated or ‘captured’ at the respective poles in the future forespore and mother cell compartments following the onset of DNA replication. In a Δ*sirA* mutant strain, 10% of sporulating cells fail to capture *oriC*. This activity is distinct from the role of SirA in DNA replication regulation, as *sirA* mutants deficient in DNA replication inhibition were able to facilitate normal *oriC* capture. Soj has also been implicated in *oriC* capture during sporulation, with 20% of cells failing to capture *oriC* in a Δ*soj* mutant strain [120,145]. There is no further increase in the failure of cells to capture *oriC* in a Δ*soj*Δ*sirA* double mutant, implying the two proteins are acting in the same pathway. Using a gain of interaction bacterial-two-hybrid screen, a potential interaction site between the C-terminus of SirA and domain III of DnaA was identified. This site overlaps with residues previously identified in the Soj-interaction site on DnaA, suggesting this interaction may facilitate *oriC* capture [145].

##### A Direct Role for Spo0A~P

The *B. subtilis* replication origin contains a number of Spo0A-boxes which partially overlap with DnaA-boxes [146] (Figure 4A). Indeed, the consensus Spo0A-box sequence 5′-TGTCGAA-3′ is similar to the DnaA-box consensus sequence 5’-TGTGNATAA-3’ [147]. Spo0A~P has been shown to bind these Spo0A-boxes in vitro [147], and sequence changes that alter the resemblance to the Spo0A-box consensus, without affecting that to the DnaA-box consensus, affect Spo0A~P, but not DnaA binding to the origin [146]. The binding of Spo0A~P to *oriC* appears to play a role in chromosome copy number control in a Δ*sda*/Δ*sirA* mutant strain when sporulation is induced by starvation, or in cells induced to sporulate during rapid growth [146]. Δ*sda* and Δ*sirA* strains each show a more significant loss of copy number control than upon mutation of the Spo0A boxes, with the phenotype being more profound for Δ*sda* than Δ*sirA* [146].

## 4. In E. coli

### 4.1. Regulatory Inactivation of DnaA (RIDA)

In *E. coli*, the concentration of available ‘initiation-active’ DnaA–ATP is considered to be a limiting factor in the initiation of DNA replication from *oriC* [148]. Thus regulation of the availability of DnaA–ATP serves to control initiation events. The ‘regulatory inactivation of DnaA’ is a term given to the process of converting ‘active’ DnaA–ATP into ‘inactive’ DnaA–ADP by ATP hydrolysis. Hydrolysis is promoted by the protein Hda, and requires a complex between DnaA, Hda and the DNA-bound polymerase β-clamp, DnaN [149,150,151]. In this way, the regulation of initiation is coupled to elongation in DNA replication, as ATP hydrolysis becomes activated following the start of DNA synthesis [150].

Hda is homologous to DnaA domain III, with a 48% sequence similarity between their AAA+ ATPase domains [150]. Hda binds DnaN via an N-terminal clamp binding motif of sequence QL[SP]LPL [152], whilst Hda:DnaA interactions are mediated via their respective ATPase domains [153]. Strains carrying *hda* deletions, and inactivated Hda mutants or DnaA mutants unable to hydrolyse ATP, exhibit overinitiation of DNA replication and growth inhibition [48,148,149,154]. Hda-mediated hydrolysis of DnaA–ATP to DnaA–ADP requires ADP-bound Hda [155] which is monomeric. In contrast, apo-Hda appears to form homodimers and larger multimers [155] implying that Hda’s oligomerisation state plays a role in its ability to promote DnaA–ATP hydrolysis [155]. A crystal structure of *Shewanella amazonensis* Hda bound to the nucleotide CDP also revealed a dimer, however because the DnaN binding motif was buried, it was assumed to represent an inactive conformation of the protein [156].

**Figure 11:**
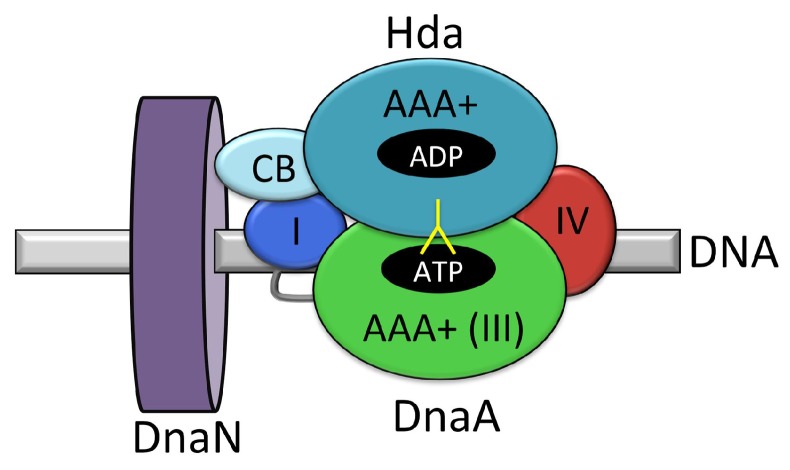
Schematic representation of the interaction of Hda with DnaA and the β-clamp. Hda-ADP (light and dark cyan) makes contacts with domain I (blue) of DnaA–ATP principally through its clamp binding domain (CB), and with domains III (green) and IV (red) of DnaA through its AAA+ domain. An arginine finger from Hda (yellow) projects into the ATP-binding pocket of DnaA and facilitates ATP hydrolysis as part of the regulatory inactivation of DnaA (RIDA). The DNA-bound β-clamp is shown in purple. Figure adapted from [77].

Mutations in both Hda and DnaA suggest that the proteins interact via their respective AAA+ domains, with the arginine finger residue of Hda playing an important role in DnaA–ATP hydrolysis [153,157]. Models of the DnaA–Hda interaction suggest that it may be similar to that formed between molecules of DnaA [156,157]. Hda’s interaction with the β-clamp is important for Hda–DnaA binding, suggesting that the β-clamp alters the conformation of the Hda–DnaA interaction to promote ATP hydrolysis [157]. Recently interactions of Hda with DnaA domains I and IV have also been shown to be important for RIDA as mutations at specific DnaA domain I and IV residues lead to higher cellular concentrations of DnaA–ATP than seen in wildtype cells [77,158]. The Hda–DnaA model suggests domain IV makes contacts with Hda at a nucleotide interaction surface towards the C-terminus of Hda [158]. It has therefore been proposed that DnaA domain I interacts with the N-terminal portion of Hda’s ATPase domain, to stabilize the DnaA–Hda interaction from both sides [77] (Figure 11). Further studies of the interactions between Hda, DnaA and the β-clamp are required to fully elucidate molecular mechanism of Hda action.

The capacity of Hda to interact with DnaA and DnaN is functionally reminiscent of the *B. subtilis* regulator YabA, which also appears to couple the initiation and elongation steps in DNA replication. However, the mechanism of action of the two proteins is quite different. The proteins are structurally and mechanistically distinct. Hda influences DnaA–ATP hydrolysis in *E. coli*, while YabA has no effect on the hydrolysis of DnaA–ATP in *B. subtilis*. The latter instead appears to influence the oligomerisation of DnaA at the origin.

### 4.2. IHF and Fis

The DNA-bending proteins integration host factor (IHF) and Fis are thought to play important roles in regulating the binding of DnaA at the origin of replication [159]. Specifically, they have been shown to shape the binding of DnaA to two *cis*-acting regulatory sites on the chromosome, *datA* and DARS [160,161] (see Section 4.3 below). Loss of IHF disrupts synchronous DNA replication. Curiously, Fis has been reported to play both inhibitory and stimulatory roles in DNA replication initiation [162,163,164].

Both proteins bind to *oriC* and act in an antagonistic manner [159,165]. Binding of IHF to a specific site in the *E. coli* replication origin as shown in Figure 4B promotes binding of DnaA at DnaA-boxes within the origin [166], contributing to DnaA oligomer formation. The binding of IHF induces a bend in the DNA, which is proposed to bring the two adjacent DnaA-boxes into closer proximity, facilitating the extension of the helical DnaA oligomer [65]. Fis has been reported to inhibit DNA unwinding at *oriC* by blocking binding of both DnaA and IHF [159]. Increasing concentrations of DnaA were found to relieve Fis inhibition, and IHF was found to redistribute DnaA molecules at *oriC* [159,166].

### 4.3. DnaA-Box Sequences: datA and DARS

In stark contrast to the clusters of DnaA-box sequences of *B. subtilis*, which do not play a significant role in replication initiation, *E. coli* possesses three loci with DnaA-box motifs that are used to regulate DNA replication initiation.

One such locus, *datA*, ≈1 kb in length and located at 94.7 min on the *E. coli* chromosome [167], contains five DnaA-boxes with high affinity for DnaA. It acts as a negative regulator of DNA replication initiation; deletion of *datA* or mutations of the DnaA-boxes within *datA* causes over-initiation of DNA replication [167,168,169]. Binding of IHF to *datA* promotes DnaA-binding and is essential for the regulatory action of *datA* [170]. *datA* had been suggested to act as a sink titrating DnaA away from the replication origin [167,168,169,170]. A recent study however, has revealed that *datA* promotes the hydrolysis of DnaA–ATP to DnaA–ADP [161], in a manner that is dependent on both IHF binding to *datA* and the DnaA arginine finger residue (Arg285). This implies that *datA* promotes the formation of a nucleoprotein complex, somewhat reminiscent of that formed at *oriC*, and stimulates the hydrolysis of DnaA–ATP at this site [161]. The binding of IHF to *datA* takes place immediately after initiation, providing a mechanism for the timing of *datA* mediated DnaA–ATP hydrolysis [161].

Two other DnaA-binding loci in *E. coli*, termed DARS1 and DARS2 for DnaA reactivating site 1 and 2, respectively, have been implicated in the reactivation of DnaA by promoting nucleotide exchange, generating DnaA–ATP from DnaA–ADP [171]. Located at 17.5 min and 64 min on the chromosome, respectively, deletion of DARS sequences causes inhibition of DNA replication due to a decrease in the cellular DnaA–ATP concentration [171]. DnaA–ADP molecules have been shown to assemble on DARS1 promoting the regeneration of DnaA–ATP [171]. The simultaneous binding of the DNA bending proteins IHF and Fis to DARS2 has been shown to facilitate DnaA–ATP regeneration in vivo [160], providing a mechanism for the timing of DnaA reactivation. The binding of IHF to DARS2 appears to be cell-cycle regulated and independent of DNA replication, whilst the binding of Fis is linked to growth phase: occurring during exponential growth but not stationary phase [160]. The role of Fis at DARS2 is consistent with a report that Fis is required for the stimulation of replication initiation in rapidly growing cells [164].

The chromosomal positioning of *datA*, DARS1 and, particularly, DARS2, relative to *oriC* has been shown to be important for the proper timing of DNA replication initiation. Translocation of these sites perturbs regulation of initiation [172,173]. However, relocation of *datA* and DARS1 perturbs DNA replication initiation only when they are moved in close proximity to the replication terminus or origin, respectively. In both cases, these effects could be attributed to a gene dosage effect, or decreased/increased proximity to DnaA [172]. The translocation of DARS2 however, has a more significant effect, with relocation of the site proximal to the terminus causing both decreased initiation events, and asynchronous replication. This suggests that the chromosomal location of DARS2 is important for regulating DNA replication synchrony [172,173].

### 4.4. SeqA

SeqA prevents re-initiation of DNA replication immediately after the previous round of replication has been initiated. It binds to hemimethylated GATC sites [174] in *oriC* and this serves to sequester the origin, preventing DnaA oligomer formation and transcription of the *dnaA* gene by blocking of the *dnaA* promoter [175,176,177,178]. The *E. coli* replication origin contains 11 GATC sites which are hemimethylated immediately after DNA replication has been initiated, because the newly synthesized strand is yet to be methylated whilst the parental DNA strand is methylated. SeqA bound to the hemimethylated GATC sites at *oriC* recruits further SeqA proteins. The origin is thus sequestered as long as six or more of the GATC sites are hemimethylated [179]. Sequestration of the origin persists for around a third of the cell cycle, after which time a combination of SeqA dissociation and methylation of the adenosine bases in the GATC sites of the newly synthesized strand by Dam methyltransferase relieves sequestration [177,180,181]. Interestingly, SeqA has also been implicated in faithful chromosome segregation [182].

### 4.5. DiaA (and HobA)

DiaA is a positive regulator of DNA replication initiation in *E. coli* influencing the frequency and timing of the initiation event [56]. It functions by binding to domain I of DnaA and promoting the oligomerisation of DnaA at the origin [82,183,184]. HobA, an orthologue of DiaA in *H. pylori* [185], is an essential regulator of DNA replication in this organism [55]. DiaA and HobA are tetramers. The structure of a HobA–DnaA domain I complex revealed a 4:4 stoichiometry, with each HobA protomer bound to one DnaA domain I [82]. DiaA has been shown to bind an equivalent site on DnaA [76], although HobA and DiaA are not interchangeable in vivo due to differences in their cognate DnaA domain I sequences [183]. Heterologous complexes can be achieved however with hybrid DnaA molecules. Thus DiaA from *E. coli* can interact with a chimaeric protein resulting from fusion of DnaA domain I from *E. coli* and DnaA domains II-IV from *H. pylori*. This confirms that DiaA and HobA are functional homologs, each promoting DnaA binding at the origin, albeit with different dynamics. HobA accelerates DnaA binding, whilst DiaA decreases the DnaA binding rate [183]. Structural and mutational studies with HobA have led to the suggestion that the tetramers function as molecular scaffolds which promote the formation of DnaA oligomers at *oriC*, however direct experimental evidence is still required [186]. DiaA may play a role in regulating the timing of helicase loading in *E. coli* as both proteins appear to bind to an overlapping site on DnaA, and DiaA has been shown to inhibit helicase loading in vitro [76].

### 4.6. Lysine Acetylation of DnaA

A recently discovered mechanism for controlling DNA replication initiation in *E. coli* involves the reversible acetylation of lysines within DnaA. Acetylation sites were identified on 13 lysines within natively expressed DnaA, including a key lysine (Lys178) required for the binding of ATP [187]. The acetylation of this residue was growth phase dependent, with peak levels observed in stationary phase. Mutation of Lys178 to Gln or Arg prevented ATP-binding to DnaA, suggesting acetylation of Lys178 would have a similar effect in inactivating the initiator. It is attractive to consider that similar, as yet unidentified, post-translational modifications may exist in other species providing an elegant mechanism for coupling DNA replication initiation to growth phase [187].

## 5. Regulatory Mechanisms for DNA Replication Regulation: *B. subtilis* vs. *E. coli*

**Figure 12:**
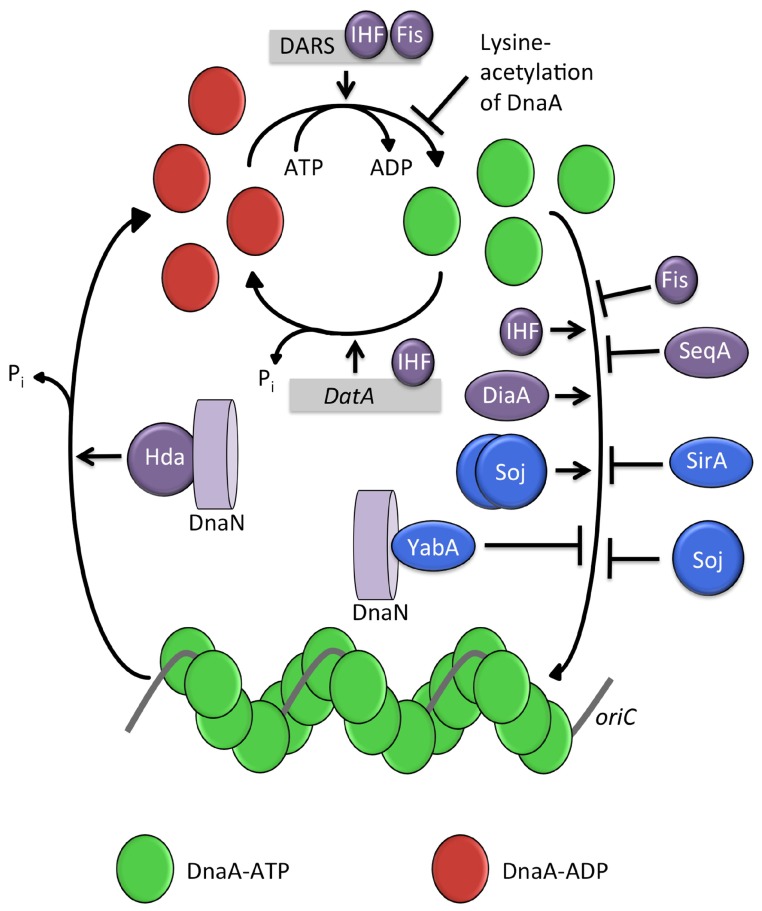
Schematic representation of the mechanisms regulating DNA replication initiation in *E. coli* and *B. subtilis*. Key regulators in *E. coli* influence the adenosine nucleotide bound state of DnaA, whilst those in *B. subtilis* influence the binding of DnaA–ATP to *oriC.* For *E. coli*, the protein regulators are shown in purple, and the DNA binding sites, DatA and DARS, are shown in grey. For *B. subtilis*, protein regulators are shown in blue. Pointed arrows indicate positive effects upon a process, and blunt ended arrows indicate inhibitory effects.

In recent years, opposing themes have emerged in the regulation of DNA replication initiation in *E. coli* and *B. subtilis* (Figure 12). It has long been recognized that the cellular DnaA–ATP concentration plays an important role in the regulation of *E. coli* replication initiation [1,4,188]. Many of the regulatory mechanisms in *E. coli* have been found to influence the adenosine nucleotide bound state of DnaA. Hda, along with the DNA polymerase clamp, DnaN, acts to promote the hydrolysis of DnaA–ATP after initiation [150]. Meanwhile, the *datA*, DARS1 and DARS2 loci influence available DnaA–ATP levels by promoting ATP-hydrolysis (*datA*) [161] or nucleotide exchange from ADP to ATP (DARS) [171]. The DNA-binding proteins Fis and IHF influence DnaA binding at these sites so as to control the cellular DnaA–ATP concentration [159,160]. Finally, lysine acetylation of DnaA coordinated with growth cycle is also believed to affect ATP-binding to DnaA and thus inhibit initiation of DNA replication in later growth phases [187].

In *B. subtilis*, by contrast, no regulator has been identified which affects the conversion of DnaA–ATP to DnaA–ADP. Instead, replication regulators in *B. subtilis* appear to act directly on the binding of DnaA to *oriC*. YabA is able to inhibit DnaA oligomer formation in vitro [112] and to affect the cooperativity of DnaA–ATP binding at *oriC* [73,122]. Monomeric Soj appears directly to inhibit DnaA oligomer formation on DNA, whilst dimeric Soj seems to be able to promote this oligomerisation [72]. Despite the fact that Soj and YabA interact with the ATPase domain of DnaA, neither protein has an effect on ATP hydrolysis in DnaA–ATP [72,112]. Instead both appear to target the DnaA oligomerisation determinants residing in domain III. Furthermore, DnaA-box clusters with significant roles in the regulation of DNA replication initiation have not been identified in *B. subtilis* [123], in marked contrast to *E. coli*. Together this evidence suggests that the primary mechanisms of DNA replication control are different in *B. subtilis* and *E. coli*. The initiation regulators determine the ligation status (ATP versus ADP) of DnaA in *E. coli* while in *B. subtilis* they act to control the downstream event of DnaA oligomerisation at *oriC*.

Despite this, a number of parallels can be drawn between the regulatory mechanisms in the two species. Both organisms utilize a major regulator during vegetative growth which interacts with both DnaA and DnaN; YabA in *B. subtilis* [110] and Hda in *E. coli* [152]. This may provide the respective species with a mechanism for appropriately timing initiation of replication, since DnaN is a key component of the DNA elongation complex. Both organisms have regulators which are implicated in chromosome segregation, SeqA in *E. coli*, and Soj and SirA during growth and sporulation of *B. subtilis*, respectively. Both organisms appear to utilize a method of origin sequestration to prevent DnaA binding: in *E. coli*, SeqA binds to newly replicated origins, and in *B. subtilis* Spo0A~P is able to bind to the origin, playing an albeit more modest role in inhibiting DNA replication. Furthermore, both organisms have evolved a regulator which targets a structurally equivalent location on DnaA domain I—the sporulation inhibitor of replication in *B. subtilis*, SirA, and the promoter of DNA replication initiation in *E. coli*, DiaA. Thus, these may represent common themes of replication regulation across bacterial species.

## Figures and Tables

**Table 1:** The essential DNA replication initiation machinery of *Bacillus subtilis* and *Escherichia. coli*.

Role in DNA Replication Initiation	*B. subtilis*	*E. coli*
Initiator	DnaA	DnaA
Helicase	DnaC	DnaB
DNA Remodelling	DnaB, DnaD	_
Helicase Loader	DnaI	DnaC
Primase	DnaG	DnaG
